# Heparin binding VEGF isoforms attenuate hyperoxic embryonic lung growth retardation via a FLK1-neuropilin-1-PKC dependent pathway

**DOI:** 10.1186/1465-9921-15-32

**Published:** 2014-03-19

**Authors:** Americo E Esquibies, Anil Karihaloo, Susan E Quaggin, Alia Bazzy-Asaad, Lloyd G Cantley

**Affiliations:** 1 Department of Pediatrics Section of Respiratory Medicine, Yale University School of Medicine, 333 Cedar Street, P.O. Box 208064, New Haven, CT 06520, USA; 2 Department of Internal Medicine Section of Nephrology, Yale University School of Medicine, 333 Cedar Street, P.O. Box 208064, New Haven, CT 06520, USA; 3 Division of Nephrology, Northwestern University Feinberg School of Medicine, 420 E. Superior St, Chicago, IL 60611, USA

**Keywords:** VEGF, Branching morphogenesis, Hyperoxia, Lung explant, Apoptosis

## Abstract

**Background:**

Previous work in our laboratory demonstrated that hyperoxia suppressed the expression of vascular endothelial growth factor (VEGF) by the embryonic lung, leading to increased epithelial cell apoptosis and failure of explant airway growth and branching that was rescued by the addition of Vegf165. The aims of this study were to determine protective pathways by which VEGF isoforms attenuate hyperoxic lung growth retardation and to identify the target cell for VEGF action.

**Methods:**

Timed pregnant CD-1 or fetal liver kinase (FLK1)-eGFP lung explants cultured in 3% or 50% oxygen were treated ± Vegf121, VEGF164/Vegf165 or VEGF188 in the presence or absence of anti-rat neuropilin-1 (NRP1) antibody or GO6983 (protein kinase C (PKC) pan-inhibitor) and lung growth and branching quantified. Immunofluorescence studies were performed to determine apoptosis index and location of FLK1 phosphorylation and western blot studies of lung explants were performed to define the signaling pathways that mediate the protective effects of VEGF.

**Results:**

Heparin-binding VEGF isoforms (VEGF164/Vegf165 and VEGF188) but not Vegf121 selectively reduced epithelial apoptosis and partially rescued lung bud branching and growth. These protective effects required NRP1-dependent FLK1 activation in endothelial cells. Analysis of downstream signaling pathways demonstrated that the VEGF-mediated anti-apoptotic effects were dependent on PKC activation.

**Conclusions:**

Vegf165 activates FLK1-NRP1 signaling in endothelial cells, leading to a PKC-dependent paracrine signal that in turn inhibits epithelial cell apoptosis.

## Background

Low oxygen tension is a persistent feature of embryonic life and is believed to stimulate lung development via transcriptional upregulation of hypoxia inducible factor dependent pathways such as Vascular endothelial growth factor A (VEGF-A) and its receptors FMS-like tyrosine kinase (FLT1 or VEGFR-1) and fetal liver kinase (FLK1 or VEGFR-2). VEGF-A is a well-characterized endothelial growth factor that is secreted by several cell types including airway epithelium and is essential for vasculogenesis and angiogenesis [1]. Both FLT1 and FLK1 have been localized to endothelial cells and are expressed throughout development. Interestingly, recent studies have shown that VEGF-A can also play a role in epithelial cell morphogenesis in both the lung and the kidney, either via an indirect effect of VEGF-stimulated vascular development on adjacent epithelial structures or a direct effect of VEGF-A on the epithelial cells themselves [2,3].

Vegf-A undergoes alternative splicing in humans to yield three major isoforms, Vegf121, 165, and 189, corresponding to the mouse isoforms VEGF120, 164, and 188 [4]. VEGF120/Vegf121 is diffusible, whereas VEGF164/Vegf165 and VEGF188/Vegf189 are variably sequestered by binding to heparan sulfate moieties on the cell surface and in the extracellular matrix. These isoforms are expressed in distinct temporo-spatial patterns, suggesting that each may serve a specific developmental function. In the mouse, VEGF164 is the most abundant during early embryonic lung development (E9.5-16), but the levels of both VEGF164 and VEGF120 decrease as development progresses, and remain relatively low in the adult lung. In contrast, VEGF188 becomes the predominant isoform after E16, remaining high throughout adulthood [5].

In prior work, we have shown that exposure of embryonic murine lung explants to hyperoxia results in downregulation of the RNA for all three major *Vegf* isoforms with an increase in airway epithelial apoptosis and marked reduction in lung bud branching and growth that is partially rescued by the addition of recombinant human Vegf165 [6]. This suggests that exogenous administration of VEGF might act therapeutically to protect against hyperoxic lung growth retardation. Both Vegf121 and Vegf165 have been tested clinically as single agents in gene therapy trials for human diseases [7-9]. However, it has become evident that the VEGF-A splice variants are not biologically equivalent or redundant [10]. For example, mice that produce VEGF120 but not VEGF164 or 188 demonstrate pruned vasculature and delayed airway development, demonstrating that VEGF164 and/or VEGF188 are required for normal airway maturation [11]. This differential function of the VEGF isoforms is in part related to their distinct downstream signaling pathways. VEGF164 and 188 utilize both the classic VEGF receptors FLT1 and FLK1, as well as the co-receptors neuropilin-1 (NRP1) and, in some cases, NRP2. These co-receptors can increase the affinity of VEGF for FLK1 and increase the downstream signaling that mediates both the mitogenic and angiogenic responses in endothelial cells [12,13]. In contrast, VEGF120/Vegf121 interacts with FLT1 and FLK1 but not NRP1, while the related protein, placental growth factor (PlGF), interacts only with FLT1 [14].

Based on these observations, we hypothesized that heparin binding VEGF isoforms might initiate specific signaling pathways and bind receptors in specific target cells in order to attenuate hyperoxic lung growth retardation. To test this hypothesis, we used either recombinant protein or adenovirus encoding 2 main lung-expressed VEGF isoforms to test the effects of re-expression of individual isoforms during hyperoxia explant culture on embryonic mouse lung growth and branching, complemented by immunofluorescence and western analysis to define the cells responding to VEGF and the downstream signaling pathways responsible for the protective effects.

We have found that both VEGF164/Vegf165 and VEGF188 can rescue lung growth under hyperoxic conditions, whereas Vegf121 cannot. Using a FLK1-eGFP transgenic mouse we show that the heparin binding VEGF isoforms activate FLK1 on developing lung endothelial cells in a NRP1 dependent manner, leading to protein kinase C (PKC) activation that mediates a paracrine signal to suppress hyperoxia-induced apoptosis in nearby airway epithelial cells. These results highlight the cross-talk between endothelial and epithelial cells during lung development as well as stress responses, and suggest that VEGF signaling via a FLK1-NRP1-PKC pathway may play an important role in prevention of hyperoxic lung growth retardation.

## Methods

### Lung explant culture

Timed pregnant CD-1 (Charles River) or FLK1-eGFP mice (Samuel Lunenfeld Research Institute, Canada) were euthanized on E10.5 or E11.5 (presence of vaginal plug was considered embryonic day 0.5) and lung buds with intact tracheas were cultured on Transwell permeable supports with DMEM + Penicillin 12.5 U/ml/Streptomycin 12.5 μg/ml (Gibco) ±10% fetal bovine serum in the bottom well. In a similar approach, we performed some experiments with E15.5 post caval lobes with tracheas instead of whole lungs since accurate quantitation of lung explants at this stage is too difficult. Explants were cultured for up to 72 hours at 37°C in sealed chambers (Billups-Rothenberg, CA) equilibrated to a humidified atmosphere of 5% CO2 with a defined oxygen concentration, balanced by nitrogen. Culture medium was replaced every 24 hours. In some experiments, human Vegf165 (R&D Systems), human Vegf121 (R&D Systems), anti-rat NRP1 antibody (R&D Systems) or GO6983 (PKC pan inhibitor, EMD Chemicals, NJ, USA) were added to the culture medium at concentrations of 50–100 ng/ml, 50 ng/ml, 10 micrograms/ml and 1–5 microM respectively as previously described [3,6,15,16]. All animal experiments were conducted under approved Institutional Animal Care and Use Committee guidelines at Yale University.

### Use of FLK1-eGFP mice

FLK1-eGFP mice expressing GFP under control of the FLK1 promoter were generated by Dr. J. Rossant (Samuel Lunenfeld Research Institute, Toronto, Ontario, Canada) and Dr M. Ema [17,18]. FLK1-eGFP mice express GFP selectively in mesodermal progenitors including hematopoietic, endothelial, cardiac and skeletal muscle precursors [17], with endothelial cells expressing eGFP beginning at the primitive streak stage (personal communication, S.E.Q.). Our immunofluorescence studies in explanted E11 (not shown)-15.5 FLK1-eGFP lungs demonstrated that all GFP + cells colocalized with the endothelial marker CD31 (Pecam-1) (Additional file 1: Figure S1).

### Quantification of lung branching

Explants were randomly assigned to 3% oxygen (to mimic in vivo lung oxygenation) or 50% oxygen culture (a concentration that we have previously shown to cause inhibition of VEGF expression accompanied by suppression of explant growth [6]) with or without added recombinant Vegf165, recombinant Vegf121, anti-rat NRP1 antibody or GO6983. Other experiments utilized infection of explants with an adenovirus encoding either DsRed (control), VEGF164 or VEGF188 for 2 days in 3% oxygen, followed by exposure to 50% oxygen. Lungs were photographed at 0, 24, 48 and 72 hours (after initiation of hyperoxia). The images were utilized to determine the number of terminal bud branches (structures with blind ends) and to quantitate total branch length (sum of straight lines beginning at the point of branching and ending at the branch tip). Each experiment utilized 9–10 explanted lungs with 2–3 lungs in each experimental group which were averaged and counted as an n of 1, and was repeated on at least 3 occasions.

### Cell apoptosis assay

Analysis of apoptosis was performed using TUNEL In Situ Cell Death Detection Kit (Roche) and cleaved caspase 3 (Asp175) antibody (Alexa Fluor 488 or 594) (1:100, Cell Signaling). Sections were counterstained with DAPI. A minimum of 12 views of 3–4 airway structures were analyzed for each explant and at least 3 explants were analyzed per experiment (averaged to comprise an n of 1 for each experiment) with a total of 5 experiments per condition. Cells were included only if they were contained within the airway branch and were defined as apoptotic only if the entire nucleus was stained by TUNEL or cytoplasm was stained by cleaved caspase 3 antibody. Over 2000 cells were counted for each condition. The total apoptosis index was defined as the percentage of TUNEL or cleaved caspase 3 positive cells/total number of cells in the airway structure (DAPI stained nuclei). Other apoptosis indices were a) endothelial compartment: total number of functional GFP positive expression and TUNEL positive cells/total number of GFP positive, b) non endothelial (mesenchymal and epithelial) compartment: total number of GFP negative TUNEL positive cells/total GFP negative DAPI positive cells or c) epithelial compartment: total number of TUNEL positive or cleaved caspase 3 positive cells within the epithelial compartment (identified by cytokeratin staining)/Total cytokeratin positive cells.

### Adenoviral infection of embryonic lung explants

Lung explants were harvested from E10.5 mouse embryos and cultured under 3% oxygen for 6 hours in 10% fetal bovine serum and Penicillin 12.5 U/ml/Streptomycin 12.5 μg/ml (Gibco) to allow for adhesion to the Transwell permeable supports. Explants were then starved and infected with 150 adenoviral particles per cell (an E10.5 mouse lung explant has approximately 15,000 cells by our count) of either *Ad-Vegf164*, *Ad-Vegf188*; or *Ad-DsRed* (a kind gift from Dr. Frank Giordano. Yale University [10]) for 2 days to allow for protein expression. Subsequently, explants were cultured in oxygen levels of 3% or 50% for another 2 days. Lungs were imaged using Hoffman contrast and fluorescence microscopy. VEGF isoform expression was quantified by real time PCR normalized to Glyceraldehyde 3-phosphate Dehydrogenase (GAPDH) expression and reported as 2-dCt. The primers were: Vegf-A164 Reverse: 5′-GAACAAGGCTCACAGTGATTTTCT-3′, Vegf-A164 and Vegf-A 188 Forward: 5′-TGCAGGCTGCTGTAACGATG-3′, Vegf-A188 Reverse: 5′- CTCCAGGATTTAAACCGGGATT-3′, Gapdh Forward: 5′-TCACCAC-CA TGGAGAAGGC-3′, Gapdh Reverse: 5′-GCTAAGCAGTTGGTGGTGCA-3′

### Immunoblotting

At least twelve lung explants/condition at E11.5 or post caval lobes at E15.5 were cultured in 3% and 50% oxygen for 2 days followed by homogenization in RIPA buffer (1% Triton X-100, 1% deoxycholate, 0.1% SDS, 20 mM Tris, 0.16 M NaCl, 1 mM EGTA, 1 mM EDTA, 15 mM sodium fluoride, 1 mM phenylmethylsulfonyl fluoride, 0.5 μg/mL leupeptin), and 0.5 μg/mL pepstatin A containing Proteinase Inhibitor Complete and phosphatase inhibitor (Roche Molecular Biochemicals, IN). Lysates were centrifuged at 10,000 rpm for 30 minutes at 4°C and supernatants used as whole tissue lysates. Protein concentration was measured using the Bradford assay. Equal amounts of protein (50–70 micrograms) were separated on NuPAGE 4-12% Bis–Tris Gels and transferred to nitrocellulose, as described earlier. Western blots were performed with antibodies against p-FLK1 (Tyr 996)(1:200 Santa Cruz Biotechnology, CA), phospho-PKC (1:1000 Cell Signaling Technologies), p-Akt 1/2/3 (1:500, Santa Cruz Biotechnology, CA) and phospho-p44/42 MAPK (Erk ½) (1:1000 Cell Signaling Technologies). To ensure equal loading, membranes were re-probed with and antibody to PKC, Akt and Actin (I-19) (1:2000 Santa Cruz Biotechnology, CA). Specific bands were visualized after incubation with the respective secondary antibodies by autoradiography by using Enhanced Chemiluminescence Substrate (Western Lightning Plus-ECL, PerkinElmer Inc, MA).

### Densitometry measurements

Densitometry measurements of Western blots from each experimental group were obtained (n = 5 for each group), and absolute values were normalized to actin.

### Immunostaining analysis

Lung explants were pooled and placed in tissue freezing medium and cut in semi-thin serial frozen sections (0.8-1 μm). Sections were washed in PBS followed by antigen retrieval (heating to 90°C for 3–10 minutes). Lungs were washed × 3 in PBS and non-specific binding blocked by incubation in 10% goat serum, 0.1% bovine serum albumin (BSA) and PBS for 1 hour. Sections were incubated overnight at 4°C with mouse anti-keratin pan monoclonal antibody (1:100 Millipore), phospho-VEGF receptor 2 (p-FLK1) (Tyr 1175) (1:200 Cell Signaling), cleaved caspase 3 (Asp 175) antibody (Alexa Fluor 488 or 594) (1:100 Cell Signaling) or purified rat anti mouse CD31 (Pecam BD Pharmingen 1:50) in 10% goat serum, 0.1% BSA and PBS solution, then washed × 3 and incubated with goat anti-mouse antibody, biotinylated goat anti-rabbit antibody or goat anti rat conjugated to Alexa 594 (1:200). Airway analysis was similar to Methods for Cell apoptosis assay. Cells were included only if they were contained within the airway branch and were defined as positive if the entire membrane was stained by p-FLK1. Over 2000 cells were counted for each condition. The Total p-FLK1 index was defined as the percentage of p-FLK1-positive cells/total number of cells in the airway structure and the p-FLK1 endothelial index was defined as the percentage of p-FLK1-positive, GFP positive cells/total number of GFP positive cells.

### Statistical analysis

All data are expressed as means ± SD. Paired Student’s t test or one-tailed Student’s t test were used for calculating statistical significance, where appropriate. A p < 0.05 was considered statistically significant.

## Results

### Heparin-binding VEGF isoforms partially rescue lung growth in hyperoxic explant culture

**Figure 1:**
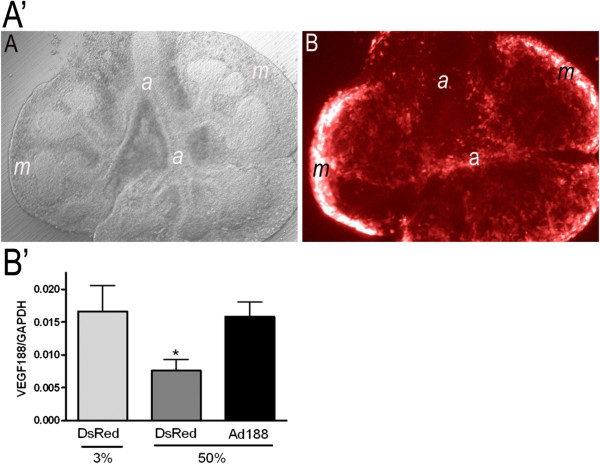
***Ad-Vegf188 *rescues hyperoxia suppression of *Vegf188 *mRNA expression. A’)** Adenoviral control infection (*Ad-DsRed*) in mouse explanted embryonic lung. **(A)** E12.5 explanted lung infected with adenovirus (Hoffmann image). **(B)** Same lung under immunofluorescence. Photographs at 10x magnification. a: airway, m: mesenchyme. **B’)** E10.5 lung explants were harvested, infected with either *Ad-DsRed* control adenovirus or *Ad-Vegf188* and then exposed at E12 to either 3% or 50% oxygen for 2 d. Quantitative PCR of *Vegf188* was performed with *Gapdh* used as control. n = 4, *p <0.05 vs. 3% alone and 50% oxygen + *AdVegf188* at E12 + 2.

We have previously shown that all VEGF isoforms are downregulated and lung development severely impaired following explant exposure to 50% oxygen. Since VEGF188 is the predominantly expressed VEGF isoform in the developing lung, we compared the ability of VEGF188 and VEGF164 to rescue lung explants from hyperoxic injury using an adenoviral expression system [10]. Infection with a control adenovirus expressing intracellular Ds-Red revealed that transgene expression is most prominent in the mesenchymal cells surrounding the developing airways (Figure 1A’). Titration of the *Ad*-*Vegf188* dose to 150 viral particles/cell led to restoration of *Vegf188* mRNA expression in explants cultured in 50% oxygen to the level seen following culture in 3% oxygen (Figure 1B’). Similar expression levels of mRNA for *Vegf164* were found following infection with this titer of *Ad-Vegf164* (data not shown). This pattern of expression was predicted to restore local levels of the relevant VEGF isoform at a location that would be available to both the mesenchymal and epithelial cell compartments. Of note, there was no detectable effect of control adenoviral infection on airway branching or branch length at 48 hours after explant culture.

**Figure 2:**
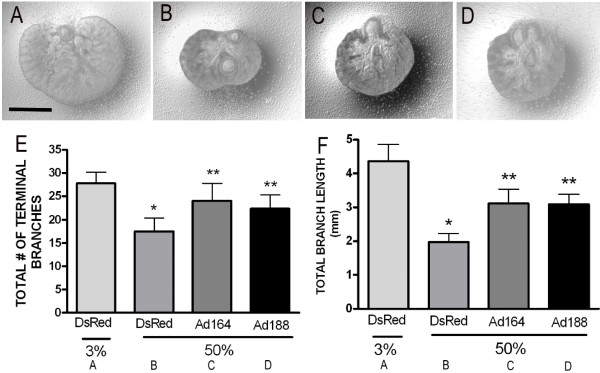
**Hyperoxia-dependent inhibition of lung morphogenesis is attenuated by VEGF164 and VEGF188.** E10.5 lung explants were harvested, infected with *Ad-DsRed***(A,B)**, *Ad-Vegf164***(C)**, or *Ad-Vegf188***(D)** and then exposed at E13 to either 3% **(A)** or 50% **(B-D)** oxygen for 2 d. **(E)** Total number of terminal branches and **(F)** total branch length were quantitated in explants treated as above. n =4 for each group, *p <0.05 vs. 3% alone and **p <0.05 vs. 50% oxygen + DsRed at E12 + 2. Photographs at 4x magnification. Bar = 1 mm.

Explants harvested from E10.5 mouse embryos were then infected with either *Ad-Vegf164*, *Ad-Vegf188* or *Ad-DsRed* control, and cultured at 37°C in a sealed chamber with 3% oxygen for 2 days (to allow for protein expression), followed by culture in either 3% or 50% oxygen for another 2 days. As previously described, exposure of lung explants infected with control adenovirus to 50% oxygen resulted in a marked decrease in airway branch numbers and branch length as compared to those cultured in 3% oxygen (Figure 2A,B, quantified in E,F). Infection with either *Ad-Vegf188* or *Ad-Vegf164* followed by culture in 50% oxygen partially rescued the decrease in branch numbers and total branch length (Figure 2C,D*)*, comparable to the rescue that we previously observed with recombinant Vegf165 [6].

### Vegf121 fails to rescue explant lung growth under hyperoxic culture

**Figure 3:**
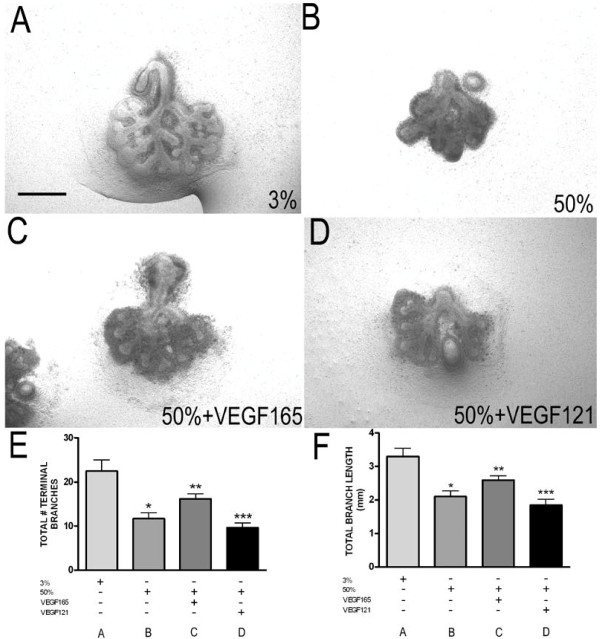
**Hyperoxia-dependent inhibition of lung morphogenesis is attenuated by Vegf165 but not Vegf121.** E11.5 lung explants exposed to **(A)** 3% oxygen **(B)** 50% oxygen **(C)** 50% oxygen + Vegf165 (50 ng/mL) or **(D)** 50% oxygen + Vegf121 (50 ng/mL ) for 2 d. **(E)** total number of terminal branches and **(F)** total branch length from lungs cultured as above were quantitated at E11.5 + 2. n = 4 per group, *p <0.05 vs. 3% alone and **p <0.05 vs. 50% alone and ***p <0.05 vs. 50% + Vegf165 at E11.5 + 2. Photographs at 4x magnification. Bar = 1 mm.

In addition to the heparin-binding VEGF isoforms 164 and 188, non-heparin binding VEGF120 is also expressed in the developing lung and suppressed by hyperoxic culture. To test the ability of this isoform to rescue lung bud growth under hyperoxic conditions, explants from E11.5 mouse embryos were harvested and cultured in: a) 3% oxygen alone, b) 50% oxygen alone, c) 50% oxygen + recombinant human Vegf165 (50 ng/ml daily), or d) 50% oxygen + recombinant human Vegf121 (50 ng/ml daily) at 37°C in a sealed chamber for 2 days. Total number of lung bud branches and total branch length were quantitated. Similar to the results with *Ad-Vegf164*, treatment with Vegf165 partially rescued lung bud branching and growth under hyperoxic conditions, whereas recombinant Vegf121 failed to rescue either lung bud branching or elongation (Figure 3). Cumulatively, these data demonstrate that the two heparin binding VEGF isoforms are equally capable of rescuing lung growth during hyperoxic explant culture, whereas VEGF120/Vegf121 appears to be insufficient to confer protection.

### Vegf165 activates FLK1 on explant GFP + endothelial cells in a neuropilin-dependent fashion

**Figure 4:**
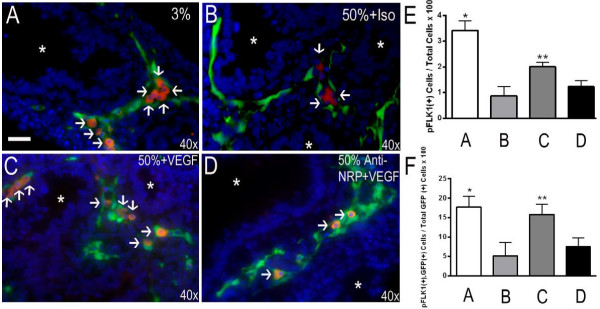
**Vegf165 activates FLK1 on GFP + endothelial cells in a NRP1-dependent manner.** FLK1-eGFP E11.5 + 2 lung explants were exposed to either 3% **(A)** or 50% **(B-D)** oxygen in the presence of either anti-NRP1 antibody (**D**, 10 microgram/ml) or isotype control **(B)** for 30 minutes, followed by Vegf165 (**C**,**D**, 100 ng/ml) for 15 min. Explants were immunostained for phosphorylated FLK1 (pFLK1). Arrows indicate pFLK1 positive (red), GFP positive (green) endothelial cells. **E)** Quantitation of pFLK1 positive cells/total cells x 100 and **F)** Quantitation of pFLK1 positive, GFP positive cells/total GFP positive cells x 100 respectively. n = 4 separate experiments with 2–3 lungs/condition/experiment. *p <0.05 vs. 50% + Isotype and 50% + Anti-NRP1 + Vegf165. **p <0.05 vs. 50% + Isotype and 50% + Anti-NRP1 + Vegf165. Photographs at 40x magnification. Bar = 20 microns. White asterisks show airway lumen.

Neuropilin-1 (NRP1) serves as a VEGF co-receptor that enhances VEGF164/Vegf165 and VEGF188/Vegf189 signaling via VEGF2 (FLK1), whereas VEGF120/Vegf121 does not utilize NRP1 for signaling. To determine which cells in the lung explant are responding to heparin-binding VEGF isoforms, and whether that response is dependent on NRP1, lung explants were isolated from FLK1-eGFP mice that express GFP in CD31+ endothelial progenitor cells (Additional file 1: Figure S1, [18]). FLK1-eGFP E11.5 lung explants were cultured for 2 days in either 3% or 50% oxygen ± recombinant Vegf165 with either anti-NRP1 or isotype control, followed by fixation and immunostaining for phospho-FLK1 and detection of GFP by fluorescence microscopy. Explants cultured in 3% oxygen without exogenous Vegf165 demonstrated pFLK1 staining selectively in GFP + cells (Figure 4A, quantified in E,F). Explants exposed to 50% oxygen demonstrated a marked reduction in pFLK1 staining (Figure 4B), consistent with the reduction in VEGF expression in those lungs [6]. The addition of exogenous Vegf165 partially rescued FLK1 activation in GFP + cells of explants cultured in 50% oxygen, whereas Vegf165 failed to activate FLK1 in the presence of the NRP1 blocking antibody (Figure 4C, D, quantified in E, F). These data demonstrate that VEGF predominantly activates FLK1 on GFP + endothelial cells, and that this activation requires NRP1.

**Figure 5:**
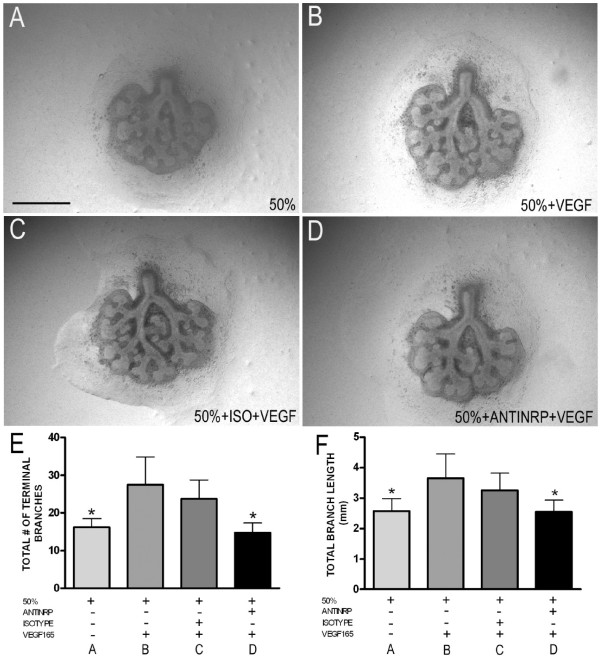
**Vegf165 attenuation of lung growth retardation requires NRP1.** E11.5 + 2 lung explants were cultured for 2 d. under the indicated conditions and total number of terminal branches and total branch length quantitated. **(A)** 50% oxygen alone **(B)** 50% oxygen + 100 ng/ml of Vegf165 **(C)** 50% oxygen + isotype control antibody for 30 minutes and then 100 ng/ml of Vegf165 **(D)** 50% oxygen + anti neuropilin-1 antibody (anti-NRP1, 10 microgram/ml) for 30 minutes and then 100 ng/ml of Vegf165 **(E)** total number of terminal branches and **(F)** total branch length from lungs cultured as above. n = 4, *p <0.05 compared to 50% oxygen + Vegf165. Photographs at 4× magnification. Bar = 1 mm.

To determine the importance of NRP1-FLK1 activation in the phenotypic response to VEGF during hyperoxia, E11.5 lungs were cultured in either 3% or 50% oxygen ± recombinant Vegf165 with either anti-NRP1 antibody or isotype control. Quantification of total terminal branches and total branch length in these experiments revealed that inhibition of FLK1 activation via the neutralizing antibody to NRP1 completely blocked the protective effect of recombinant Vegf165 addition to explant growth in the setting of hyperoxia (Figure 5A-D, quantified in E, F).

### Heparin-binding VEGF isoforms prevent hyperoxia-induced apoptosis in airway epithelial cells

**Figure 6:**
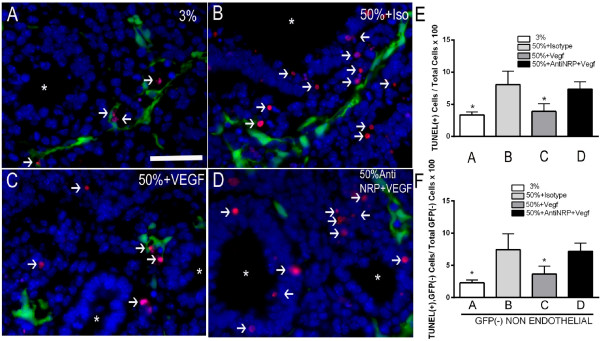
**Vegf165 attenuated hyperoxia-induced apoptosis in non-endothelial cells.** FLK1-eGFP E11.5 lungs were harvested and exposed for 2 d. to **(A)** 3% oxygen, **(B)** 50% oxygen + isotype control, **(C)** 50% oxygen + Vegf165 (100 ng/ml) and **(D)** 50% oxygen + anti-NRP1 (10 microgram/ml) + Vegf165 (100 ng/ml), followed by staining for TUNEL (red) and DAPI (blue). Endothelial cells fluoresce green (GFP). Arrows indicate TUNEL positive cells. **(E)** Quantitation of TUNEL positive cells/total cells x 100 and **(F)** TUNEL positive, GFP negative cells/total GFP negative cells × 100. n = 4 separate experiments with 2–3 lungs/condition/experiment. *p <0.05 vs. 50% and 50% + Anti-NRP1 + Vegf165. Bar = 20 microns. Photographs at 40× magnification. White asterisks point airway lumen.

**Figure 7:**
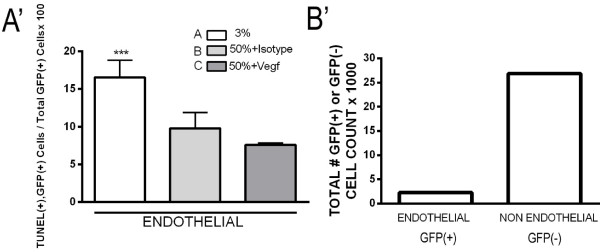
**Hyperoxia suppresses endothelial cell apoptosis.** FLK1-eGFP E11.5 lungs were harvested and exposed for 2 d. to **(A)** 3% oxygen, **(B)** 50% oxygen + Isotype, **(C)** 50% oxygen + Vegf165 (100 ng/ml). **(A’)** Quantitation of TUNEL positive, GFP positive cells/total GFP positive cells. ***p <0.05 vs. 50% + Isotype and 50% + Vegf165. **(B’)** Total number of GFP positive (endothelial) cells and GFP negative (non-endothelial) cells counted for this experiment in thousands during exposure to 3% oxygen.

The functional role of NRP1-FLK1 activation in VEGF-mediated protection from hyperoxic lung injury was analyzed using TUNEL staining of FLK1-eGFP explants cultured for 2 days under 3% oxygen, 50% oxygen, 50% oxygen + Vegf165 or 50% oxygen + anti-NRP1 + Vegf165. Quantitation of total TUNEL positive cells/DAPI-positive nuclei demonstrated that Vegf165 decreased the number of apoptotic cells in hyperoxia treated explants, and that this protective effect was prevented by the NRP1 blocking antibody (Figure 6A-D, quantified in E). The overall increase in apoptosis seen following hyperoxic explant culture is predominantly due to an increase in the GFP negative cells (non-endothelial cell compartment). These cells did not have detectable FLK1 activation in response to Vegf165 (Figure 4), yet comprised the majority of cells that exhibited the Vegf165/NRP1-dependent reduction in apoptosis (quantified in 6 F). Interestingly, endothelial cells (defined as GFP positive cells outside of the airway basement membrane) exhibited a VEGF independent decrease in apoptosis when exposed to hyperoxia that was not significantly affected by the addition of Vegf165 (Figure 7A’). These cells made up less than 10% of all explant cells (Figure 7B’) and thus did not significantly impact the overall apoptotic rate.

**Figure 8:**
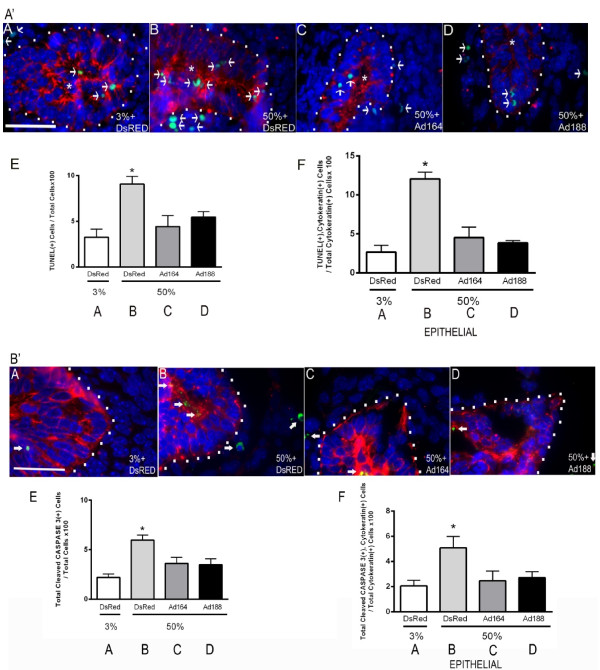
**VEGF164 and VEGF188 decrease hyperoxia-induced apoptosis in airway epithelial cells. (A’)** E10.5 lungs were harvested, infected with *Ad-DsRed***(A,B)**, *Ad-Vegf164***(C)** or *Ad-Vegf188***(D)** and exposed on E12.5 to either 3% **(A)** or 50% **(B-D)** oxygen for 2 d. followed by TUNEL staining (green), cytokeratin staining (red), and DAPI staining (blue). The cytokeratin positive epithelial compartment is outlined with white dots. Arrows indicate TUNEL positive nuclei (green). **(E)** Quantification of TUNEL positive cells/total cells and **F)** TUNEL positive, cytokeratin positive cells/total cytokeratin positive cells. **(B’)** Explant lungs treated as in **(A’)** with similar conditions **(A-D)** were stained with anti-cleaved caspase 3 (green), cytokeratin (red) and DAPI (blue). Arrows indicate cleaved caspase 3 positive cells. **(E)** Quantification of cleaved caspase 3 positive cells/total cells and **F)** Cleaved caspase 3 positive, cytokeratin positive cells/total cytokeratin positive cells. n = 4 separate experiments with 2–3 lungs/condition/experiment. *p < 0.05 3% oxygen + DsRed, 50% oxygen + *Ad-Vegf164* or *Ad-Vegf188* vs. 50% oxygen + *Ad-DsRed*, n = 4 Bar = 20 microns. Photographs at 100× magnification.

Since VEGF188 also provided morphologic protection to explants cultured under hyperoxic conditions (Figure 2), lungs treated with either *Ad-Vegf188* or *Ad-Vegf164* were analyzed for cellular apoptotic rates in the different cellular compartments. Analysis of TUNEL staining (Figure 8A’) and cleaved caspase-3 staining (Figure 8B’) combined with cytokeratin staining to identify airway epithelial cells revealed that hyperoxia increased the rate of apoptosis predominantly in epithelial cells of lung explants (Figures 8A’A,B and 8B’A,B quantified in 8A’E,F and 8B’E,F). Infection with either *Ad-Vegf188* or *Ad-Vegf164* reduced epithelial apoptosis to rates that were indistinguishable from that seen in control explants. These data demonstrate that culture of explants in 50% oxygen suppresses the expression of heparin-binding VEGF isoforms that normally serve to activate endothelial cell NRP1-FLK1 and suppress apoptosis of nearby epithelial cells. The addition of VEGF164/Vegf165 or VEGF188 during hyperoxic culture can rescue endothelial FLK1 activation and prevent epithelial cell apoptosis.

### VEGF 165 attenuation of hyperoxic injury requires endothelial PKC pathway activation

**Figure 9:**
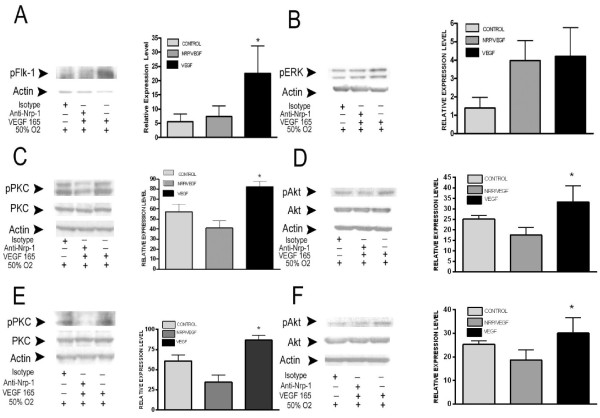
**Neuropilin-1 is required for Vegf165 activation of PKC and AKT signaling.** Lysates of E11.5 + 2 **(A-D)** and E15.5 + 2 **(E-F)** lung explants exposed to 50% oxygen ± isotype control ± anti-NRP1 (10 micrograms/ml) ± Vegf165 (100 ng/ml, 15 minutes) were western blotted for pFLK1, phospho-ERK (pERK), phospho-AKT (pAKT), AKT, pan-phospho-PKC (pPKC) and PKC with actin as loading control. Representative immunoblots are shown in **(A, B, C, D, E and F)**. Accompanying graph of n =4 experiments. *p <0.05 50% oxygen + Vegf165 vs. 50% + isotype (for pFLK1 and pPKC) and 50% oxygen + anti-NRP1 and then Vegf165 (for pFLK1, pAKT and pPKC).

Multiple laboratories have shown that activation of the ERK1/2, PI 3-K, and PKC pathways are required for tubulogenesis and/or branching morphogenesis in epithelial cells stimulated by growth factors such as HGF, epidermal growth factor and VEGF [3,19]. To determine what NRP1-dependent signaling pathway(s) mediate the protective effects of Vegf165, E11.5 (Figure 9A-D) and E15.5 (Figure 9E-F) lung explants were cultured under 50% oxygen and stimulated with Vegf165 for 15 min ± anti-NRP1. Western analysis showed that explants treated with Vegf165 had a 4-fold increase in the phosphorylation level of FLK1 as compared to lungs treated with Vegf165 in the presence of the NRP1 blocking antibody (Figure 9A). Analysis of downstream signaling pathways demonstrated that VEGF-stimulated activation of PKC and Akt were NRP1-dependent, and thus candidates for inducing the *Vegf*-stimulated protective response, whereas activation of ERK was NRP1 independent (Figure 9B-F).

**Figure 10:**
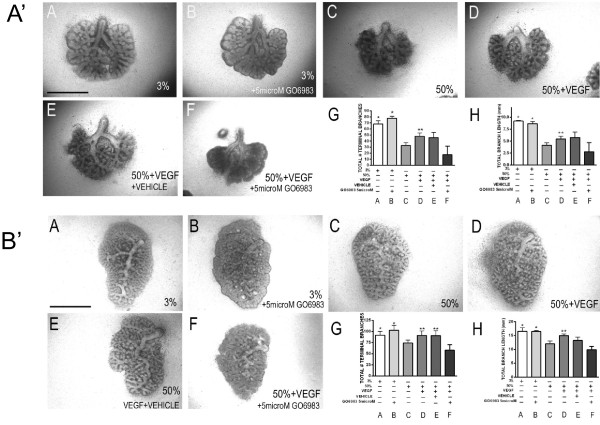
**Vegf165 requires PKC activation to maintain explant growth under hyperoxia. A’**. E11.5 whole lung explants were exposed to 3% ± GO6983 (5 μM) **(A-B)** and 50% oxygen **(C)** for 3 days in the presence of Vegf165 (100 ng/ml, **D**), Vegf165 + DMSO **(E)**, Vegf165 + GO6983 (5 μM, **F**) followed by quantification of total number of terminal branches **(G)** and total branch length **(H)**. n =5 separate experiments. *p <0.05 vs. **C**, **D**, **E** and **F**. **p <0.05 vs. **C** and **F** at E11.5 + 3. Bar = 1 mm. Photographs at 4x magnification. **B’** E15.5 lung post caval lobe explants **(B’ A-F)** were exposed to 3% ± GO6983 (5 μM) **(A-B)** and 50% oxygen **(C)** for 2 days in the presence of Vegf165 (100 ng/ml, **D**), Vegf165 + DMSO **(E)**, Vegf165 + GO6983 (5 μM, **F**) followed by quantification of total number of terminal branches **(G)** and total branch length **(H)**. n =4 separate experiments. *p <0.05 vs. **C** and F. **p <0.05 vs. **C** and **F** at E15.5 + 2. Bar = 0.5 mm. Photographs at 4× magnification.

**Figure 11:**
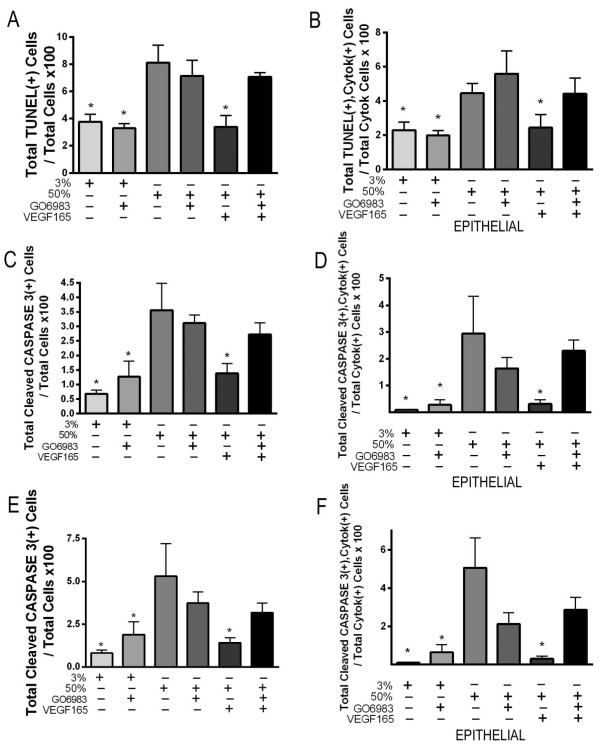
**Activation of PKC is required for Vegf-mediated anti-apoptotic responses. A-B**. E11.5 lung explants exposed to either 3% oxygen or 50% oxygen ± Vegf165 (100 ng/ml) ± GO6983 (5 μM) for 3 days and stained for TUNEL, cytokeratin and DAPI. **(A)** Quantitation of total TUNEL positive cells/total cells x 100 in explants cultured. **(B)** Quantitation of total TUNEL positive, cytokeratin positive cells/total cytokeratin positive cells. *p <0.05 vs. 50% oxygen, 50% oxygen + GO6983 and 50% oxygen + GO6983 + Vegf165 at E11.5 + 3. C-D E11.5 lung explants exposed to either 3% oxygen or 50% oxygen ± Vegf165 (100 ng/ml) ± GO6983 (5 μM) for 3 days and stained for cleaved caspase 3, cytokeratin and DAPI. **(C)** Quantitation of total cleaved caspase 3 positive cells/total cells x 100. **(D)** Quantitation of total cleaved caspase 3 positive, cytokeratin positive cells/total cytokeratin positive cells. *p <0.05 vs. 50% oxygen, 50% oxygen + GO6983 and 50% oxygen + GO6983 + Vegf165 at E11.5 + 3. E-F E15.5 Post caval lung explants exposed to either 3% oxygen or 50% oxygen ± Vegf165 (100 ng/ml) ± GO6983 (5 μM) for 2 days and stained for cleaved caspase 3, cytokeratin and DAPI. **(E)** Quantitation of total cleaved caspase 3 positive cells/total cells x 100. **(F)** Quantitation of total cleaved caspase 3 positive, cytokeratin positive cells/total cytokeratin positive cells. *p <0.05 vs. 50% oxygen, 50% oxygen + GO6983 and 50% oxygen + GO6983 + Vegf165 at E15.5 + 2 .n = 3–4 separate experiments with 2–3 lungs/ condition/experiment.

Based on our observation that Vegf165/NRP1-dependent PKC activation was more pronounced than was Akt activation, we chose to focus on the role of PKC signaling in the protection of lung explants from hyperoxic injury. Whole lung explants from E11.5 and post caval lobes from E15.5 mouse embryos were harvested and cultured in 3% or 50% oxygen ± Vegf165 ± the pan-PKC inhibitor GO6983. Treatment with GO6983 did not alter basal explant growth/branching under 3% oxygen, but prevented the Vegf165-mediated rescue of growth under 50% oxygen culture at both time points (Figure 10A’, B’). Consistent with our finding that the Vegf165-dependent rescue of explant branching and growth correlated with increased epithelial cell survival under hyperoxic conditions, the addition of GO6983 prevented the Vegf165-dependent decrease in hyperoxia-induced epithelial apoptosis as judged by both TUNEL (Figure 11A-B for explants at E11.5) and cleaved caspase 3 staining (Figure 11C-D for explants at E11.5 and 11E-F for post caval lobes at E15.5).

## Discussion

The airway is central to the defense of the lung against environmental toxins such as high oxygen concentrations or infectious pathogens. During lung development the airway epithelium is a major source of the vasculogenic factor VEGF that is believed to be critical in synchronizing the parallel development of the pulmonary vasculature and the airways [1,20]. Here we extend the scope of that epithelial-endothelial cross-talk by demonstrating that suppression of VEGF expression following exposure to hyperoxia leads to the loss of endothelial FLK1-NRP1 activation and increased epithelial apoptosis.

While all three VEGF isoforms are expressed in the developing lung, the heparin sulfate binding isoforms predominate with *Vegf164* expressed during early embryogenesis and *Vegf188* dominating after E16 [6]. Based on the phospho-FLK1 staining that we observed in control and VEGF stimulated FLK1-eGFP transgenic explants, it appears that the major target of VEGF is the endothelial cell. Furthermore, we show that functional activation of endothelial FLK1 by VEGF requires the engagement of the NRP1 co-receptor. Consistent with this, we find that only the VEGF isoforms that utilize NRP1 for maximal FLK1 activation (164/165 and 188/189) are able to confer functional protection against hyperoxic lung growth retardation.

NRP1 enhances the binding of VEGF to FLK1 and thereby increase its tyrosine kinase activity [21,22]. The endothelial cell response to this increased kinase activity has been shown to include activation of several downstream signaling pathways including the extracellular-signal-regulated kinases ERK1/2, phosphatidylinositol 3-kinase (PI 3-K) and its effector Akt, as well as PKC. Our results indicate that Vegf165 stimulates FLK1 dependent activation of all three of these intracellular pathways in the explanted lung. This activation occurs within 15 minutes of Vegf165 addition, suggesting that these signaling pathways are being activated in the phospho-FLK1 positive endothelial cells; although we cannot exclude the possibility that such activation may occur in the airway epithelium as well.

Although each of these signaling pathways has been shown under specific conditions to activate anti-apoptotic responses, we detected no difference in endothelial apoptotic rates following Vegf165 addition despite a significant increase in FLK1 activation. In fact, in contrast to the marked increase in epithelial cell apoptosis in response to hyperoxic culture, endothelial apoptosis was significantly decreased in explants exposed to hyperoxia. A similar finding made by Reyburn et al. determined that thinning of the distal airways during hyperoxia was halted in the setting of hyperoxia due to failure of apoptosis of mesenchymal cells [23]. Thus the suppression of VEGF expression and subsequent loss of endothelial FLK1 activation seen during hyperoxic culture ( [6] and Figure 4) does not appear to induce endothelial apoptosis.

These findings suggest that the mechanism of VEGF-dependent protection against epithelial apoptosis is not due to improved endothelial cell survival, but rather a FLK1-stimulated endothelial-epithelial paracrine response. Our demonstration that the Vegf165-stimulated ERK1/2 activation was not dependent on NRP1 activation, whereas the Vegf165-mediated anti-apoptotic response was dependent on NRP1 activation, led us to exclude this pathway as a likely mediator of the paracrine protective response. Instead we focused the current studies on PKC since activation of this pathway exhibited the greatest dependence of FLK1-NRP1 co-stimulation.

PKCs are serine threonine kinases that are abundantly expressed during lung development. Studies done using pharmacological PKC inhibitors and PKC gene knockout have revealed that these enzymes regulate multiple cellular responses including vascular permeability, smooth muscle contraction, cell migration, cell proliferation and apoptosis [24,25]. There is increasing evidence that VEGF-mediated activation of PKC can also induce protection against damaging insults (e.g. radiation, inflammatory diseases, hyperoxia) [16,26-28]. Our phospho-PKC antibody detected two PKC species that were activated in response to Vegf165, migrating at ranges between 80 and 90 kDa, respectively (Figure 9C-E). The higher molecular-weight band is of the predicted size of PKC ϵ, which has been shown to be a Vegf-Flk1 activation target in human endothelial cells [29]. The 80-kDa band could be one of several PKCs, including PKC α, βI, βII, γ, η, and/or θ. VEGF has been found to activate many of these isoforms in endothelial cells, including PKC α, βI, βII, and γ [30-32].

Our observation that non-endothelial cells are more protected by the VEGF-FLK2/NRP1-PKC pathway than are endothelial cells may involve activation of antioxidant defense enzymes. It has been suggested that in the premature newborn, failure to elevate the activities of these antioxidant enzymes can increase the lung damage caused by hyperoxia [33]. Peroxiredoxins belong to an antioxidant protein family with many distinct members (I-VI), with some members highly expressed in bronchial/alveolar epithelial cells [34]. Interestingly, Das et al. found that hyperoxia-mediated induction of peroxiredoxin expression in A549 cells is partially dependent on PKC signaling [35]. VEGF/PKC-dependent elevation of peroxiredoxin in the airway epithelium may therefore provide a pathway for protection against oxidative stress during exposure to higher levels of oxygen.

The identity of the VEGF-FLK1/NRP1-PKC-dependent paracrine signal is of great interest but as yet unknown. Matrix metalloproteinases (MMPs) are one attractive candidate. In endothelial progenitor cells VEGF can stimulate MMP secretion [36], and the secretion of many MMPs, including MMP1, MMP2, MMP3 and MMP9 has been found to be regulated by PKC activation [37,38]. MMPs can act to release matrix- and cell- associated growth factors such as FGF2, VEGF that can in turn stimulate anti-apoptotic effects by binding their cognate receptors on nearby cells [39,40]. Elucidation of the role of MMPs in the current endothelial-epithelial cross-talk clearly requires additional studies.

While this study has identified a novel VEGF-stimulated FLK1-NRP1 mediated mechanism of endothelial-epithelial cross-talk, there are limitations to the approach utilized that must be considered when placing this data in the larger context of *in vivo* exposure to hyperoxia. By definition, explants lack a normal circulation and all of the regulatory signals that would be derived from that circulation. For example, the expression of MMPs can be markedly altered in response to the inflammation that is seen following in vivo exposure to hyperoxia [41]. Furthermore, in order to accurately quantify morphologic responses to hyperoxia we utilized embryonic mouse lungs from E11.5 and lung lobe segments from E15.5, quite early in development as compared to most pre-term infants. The experiments at E15.5 were within the range of the canalicular stage of human lung development (17–25 weeks of gestation) and recapitulated the findings in E11.5 explants. However, we cannot rule-out a shift in the dependence on VEGF-NRP1-FLK1 signaling closer to birth. Finally, we cannot exclude the importance of the fibroblast-epithelial cross talk during branching morphogenesis since hyperoxia can induce apoptosis of the mouse lung mesenchyme in the canalicular stage [42].

## Conclusions

We propose that VEGF heparin binding isoforms normally activate FLK1-NRP1 in the hypoxic developing lung endothelium, leading to PKC activation that induces a paracrine signal to suppress epithelial apoptosis. This pathway is downregulated in hyperoxia due to suppression of VEGF164 and 188 expression. The ability of exogenously added Vegf165 to restore FLK1-NRP1-PKC activation and suppress epithelial apoptosis suggests that this pathway may provide a novel approach to decrease hyperoxic lung growth retardation. In particular, since excessive VEGF administration can induce vascular leak [43] and disrupt airway development, these data suggest that a selective activator of the specific PKC isoform involved in stimulating the paracrine signal and/or addition of the paracrine factor itself might serve as less toxic therapies.

## Abbreviations

VEGF: Vascular endothelial growth factor; FLK1: Fetal liver kinase; PKC: Protein kinase C; NRP1: Neuropilin-1; FLT1: FMS-like tyrosine kinase; PlGF: Placental growth factor; MMP: Matrix metalloproteinases; TUNEL: Terminal deoxynucleotidyl transferase dUTP nick end labeling; GFP: Green fluorescent protein.

## Competing interests

None of the authors has a financial relationship with a commercial entity that has an interest in the subject of this manuscript.

## Authors’ contributions

AEE, LGC, SEQ, AB-A and AK, conception and design; AEE, LGC and AK, data acquisition, analysis and interpretation; and all authors drafting manuscript. All authors read and approved the final manuscript.

## Supplementary Material

Additional file 1: Figure S1 CD31 endothelial marker colocalizes with FLK-eGFP cells. A-I. E15.5 Flk1-GFP lung explants are seen under fluorescence microscopy (green) (A,D and G). CD31 (red) endothelial marker is observed in B, E and H. Colocalization of GFP cells and CD31 is observed in C, F and I. Photographs at 20×, 40× and 100× magnification. Click here for file
