# Supplementary figures and images for: Heparin binding VEGF isoforms attenuate hyperoxic embryonic lung growth retardation via a FLK1-neuropilin-1-PKC dependent pathway
Source: Respir Res. 2014 Mar 19;15(1):32. doi: 10.1186/1465-9921-15-32 (PMC4004166; doi:10.1186/1465-9921-15-32)

## SUPPLEMENTAL FIGURE 1

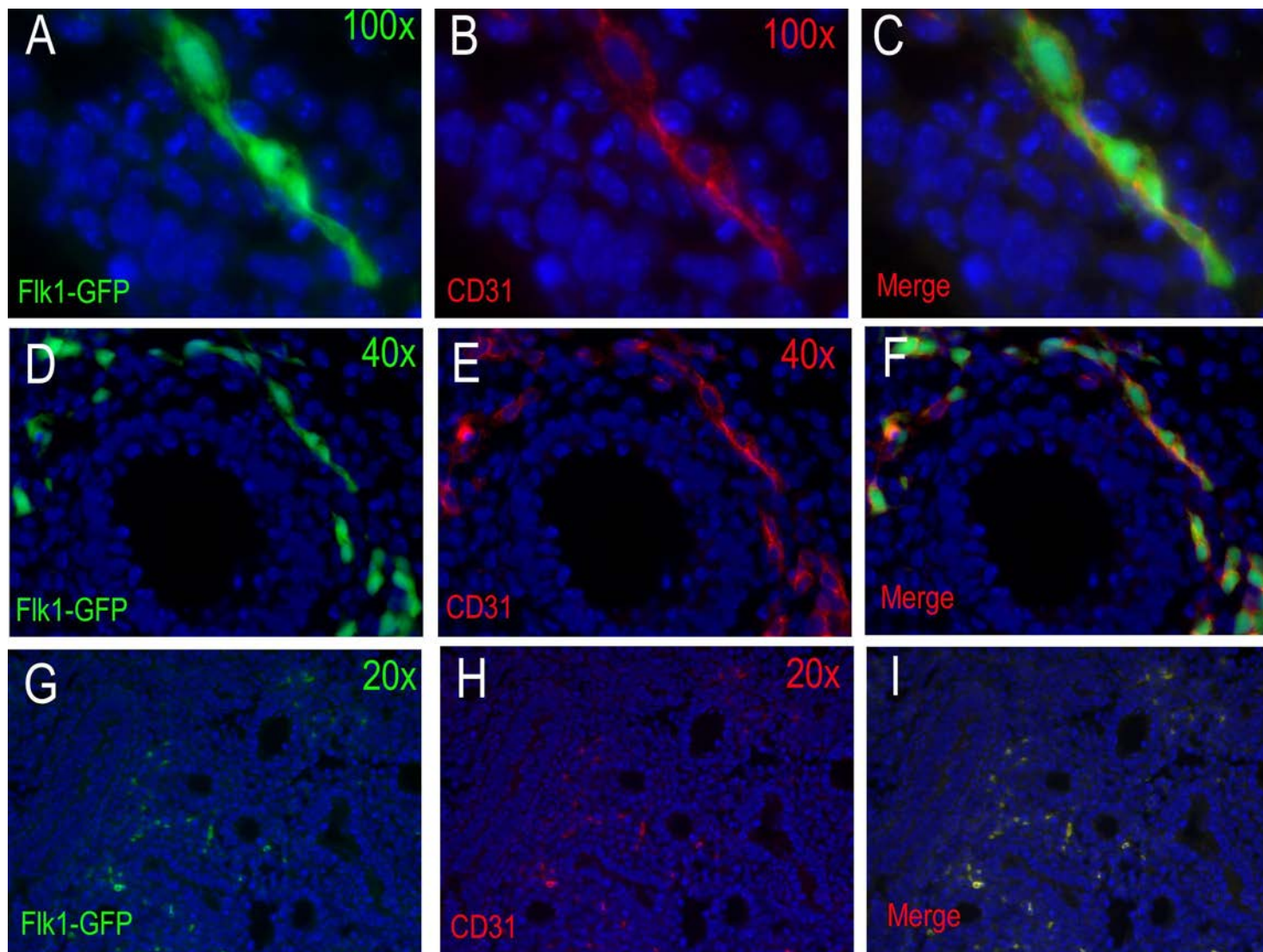

CD31 endothelial marker colocalizes with FLK1-eGFP cells

Supplement: Additional file 1: Figure S1 — CD31 endothelial marker colocalizes with FLK-eGFP cells. A-I. E15.5 Flk1-GFP lung explants are seen under fluorescence microscopy (green) (A,D and G). CD31 (red) endothelial marker is observed in B, E and H. Colocalization of GFP cells and CD31 is observed in C, F and I. Photographs at 20×, 40× and 100× magnification. [file 1465-9921-15-32-S1.pdf]
